# A systematic review of artificial intelligence chatbots for promoting physical activity, healthy diet, and weight loss

**DOI:** 10.1186/s12966-021-01224-6

**Published:** 2021-12-11

**Authors:** Yoo Jung Oh, Jingwen Zhang, Min-Lin Fang, Yoshimi Fukuoka

**Affiliations:** 1 Department of Communication, University of California Davis, Davis, USA; 2 Department of Public Health Sciences, University of California Davis, Davis, USA; 3 Education and Research Services, University of California, San Francisco (UCSF) Library, UCSF, San Francisco, USA; 4 Department of Physiological Nursing, UCSF, San Francisco, USA

**Keywords:** Artificial intelligence, Chatbot, Conversational agent, Physical activity, Weight loss, Weight maintenance, Diet, Nutrition, Sedentary behavior, Systematic review

## Abstract

**Background:**

This systematic review aimed to evaluate AI chatbot characteristics, functions, and core conversational capacities and investigate whether AI chatbot interventions were effective in changing physical activity, healthy eating, weight management behaviors, and other related health outcomes.

**Methods:**

In collaboration with a medical librarian, six electronic bibliographic databases (PubMed, EMBASE, ACM Digital Library, Web of Science, PsycINFO, and IEEE) were searched to identify relevant studies. Only randomized controlled trials or quasi-experimental studies were included. Studies were screened by two independent reviewers, and any discrepancy was resolved by a third reviewer. The National Institutes of Health quality assessment tools were used to assess risk of bias in individual studies. We applied the AI Chatbot Behavior Change Model to characterize components of chatbot interventions, including chatbot characteristics, persuasive and relational capacity, and evaluation of outcomes.

**Results:**

The database search retrieved 1692 citations, and 9 studies met the inclusion criteria. Of the 9 studies, 4 were randomized controlled trials and 5 were quasi-experimental studies. Five out of the seven studies suggest chatbot interventions are promising strategies in increasing physical activity. In contrast, the number of studies focusing on changing diet and weight status was limited. Outcome assessments, however, were reported inconsistently across the studies. Eighty-nine and thirty-three percent of the studies specified a name and gender (i.e., woman) of the chatbot, respectively. Over half (56%) of the studies used a constrained chatbot (i.e., rule-based), while the remaining studies used unconstrained chatbots that resemble human-to-human communication.

**Conclusion:**

Chatbots may improve physical activity, but we were not able to make definitive conclusions regarding the efficacy of chatbot interventions on physical activity, diet, and weight management/loss. Application of AI chatbots is an emerging field of research in lifestyle modification programs and is expected to grow exponentially. Thus, standardization of designing and reporting chatbot interventions is warranted in the near future.

**Systematic review registration:**

International Prospective Register of Systematic Reviews (PROSPERO): CRD42020216761.

**Supplementary Information:**

The online version contains supplementary material available at 10.1186/s12966-021-01224-6.

## Background

Artificial Intelligence (AI) chatbots, also called conversational agents, employ dialogue systems to enable natural language conversations with users by means of speech, text, or both [1]. Powered by natural language processing and cloud computing infrastructures, AI chatbots can participate in a broad range, from constrained (i.e., rule-based) to unconstrained conversations (i.e., human-to-human-like communication) [1]. According to a Pew Research Center survey, 46% of American adults interact with voice-based chatbots (e.g., Apple’s Siri and Amazon’s Alexa) on smartphones and other devices [2]. The use of AI chatbots in business and finance is rapidly increasing; however, their use in lifestyle modification and health promotion programs remains limited.

Physical inactivity, poor diet, and obesity are global health issues [3]. They are well-known modifiable risk factors for cardiovascular diseases, type 2 diabetes, certain types of cancers, cognitive decline, and premature death [3–6]. However, despite years of attempts to raise awareness about the importance of physical activity (PA) and healthy eating, individuals often do not get enough PA nor do they have healthy eating habits [7, 8], resulting in an increasing prevalence of obesity [9, 10]. With emerging digital technologies, there has been an increasing number of programs aimed at promoting PA, healthy eating, and/or weight loss, that utilize the internet, social media, and mobile devices in diverse populations [11–14]. Several systematic reviews and meta-analyses [15–19] have shown that these digital technology-based programs resulted in increased PA and reduced body weight, at least for a short duration. While digital technologies may not address environmental factors that constrain an individual’s health environment, technology-based programs can provide instrumental help in finding healthier alternatives or facilitating the creation of supportive social groups [13, 14]. Moreover, these interventions do not require traditional in-site visits, and thus, help reduce participants’ time and financial costs [16]. Albeit such potentials, current research programs are still constrained in their capacity to personalize the intervention, deliver tailored content, or adjust the frequency and timing of the intervention based on individual needs in real time.

These limitations can be overcome by utilizing AI chatbots, which have great potential to increase the accessibility and efficacy of personalized lifestyle modification programs [20, 21]. Enabling AI chatbots to communicate with individuals via web or mobile applications can make these personalized programs available 24/7 [21, 22]. Furthermore, AI chatbots provide new communication modalities for individuals to receive, comprehend, and utilize information, suggestions, and assistance on a personal level [20, 22], which can help overcome one’s lack of self-efficacy or social support [20]. AI chatbots have been utilized in a variety of health care domains such as medical consultations, disease diagnoses, mental health support [1, 23], and more recently, risk communications for the COVID-19 pandemic [24]. Results from a few systematic reviews and meta-analyses suggest that chatbots have a high potential for healthcare and psychiatric use, such as promoting antipsychotic medication adherence as well as reducing stress, anxiety, and/or depression symptoms [1, 25, 26]. However, to the best of our knowledge, none of these studies have focused on the efficacy of AI chatbot-based lifestyle modification programs and the evaluation of chatbot designs and technologies.

Therefore, this systematic review aimed to describe AI chatbot characteristics, functions (e.g., the chatbot’s persuasive and relational strategies), and core conversational capacities, and investigate whether AI chatbot interventions were effective in changing PA, diet, weight management behaviors, and other related health outcomes. We applied the AI Chatbot Behavior Change Model [22], designed to inform the conceptualization, design, and evaluation of chatbots, to guide our review. The systematic review provides new insights about the strengths and limitations in current AI chatbot-based lifestyle modification programs and can assist researchers and clinicians in building scalable and personalized systems for diverse populations.

## Methods

The protocol of this systematic review was registered at the International Prospective Register of Systematic Reviews (PROSPERO) (ID: CRD42020216761). The systematic review was conducted in accordance with the Preferred Reporting Items for Systematic Reviews and Meta-analysis guidelines.

### Eligibility criteria

Table 1 shows the summary of the inclusion and exclusion criteria of the study characteristics based on the PICOS framework (i.e., populations/participants, interventions and comparators, outcome(s) of interest, and study designs/type) [27]. We included peer-reviewed papers or conference proceedings that were available in full-text written in English. Review papers, protocols, editorials, opinion pieces, and dissertations were excluded.

**Table 1:** Summary of inclusion and exclusion criteria

		Inclusion criteria	Exclusion criteria
**P**	Populations/participants	Adults and/or children who use AI chatbots for PA, diet, and/or weight management	None
**I**	Interventions	Constrained^a^ and/or unconstrained^b^ text and/or speech-based AI chatbots operating as standalone software or via a web browser or mobile application	Chatbots that are part of virtual reality, augmented reality, embodied agents, and/or therapeutic robots
**C**	Comparators	With or without a usual care group^c^, comparison group, or an attention control group	None
**O**	Outcome(s)	Main outcomes: Changes in self-reported and/or objectively measured PA, sedentary behavior, diet, and/or body weight Secondary outcomes: Feasibility, acceptability, safety (e.g., adverse events, injury), and/or user satisfaction of chatbots if available	Studies that report only chatbot infrastructure or *algorithm designs*
**S**	Study designs/types	Randomized controlled trials or quasi-experimental studies	Qualitative studies, case-control studies, cross-sectional studies, or cohort studies

*AI* artificial intelligence, *PA* physical activity

^a^ Users can only respond by selecting predefined conversational lines

^b^ Users can respond freely by inputting natural conversational lines

^c^ Usual care group refers to the research group where individuals receive routine care from health care providers

### Information sources and search strategy

In consultation with a medical librarian (MF), pre-planned systematic search strategies were used for six electronic databases (PubMed, EMBASE, ACM Digital Library, Web of Science Core Collection, PsycINFO, and IEEE). A combination of MeSH/Emtree terms and keyword searches were used to identify studies on AI chatbot use in lifestyle changes; the comprehensive search strategies for each database are provided in Additional file 1. Further, hand-searching was done to ensure that relevant articles were not missed during the data collection. The searches were completed on November 14, 2020. No date limits were applied to the searches.

### Study selection

All retrieved references were imported into the Endnote reference management software [28], and duplicates were removed. The remaining references were imported into the Covidence systematic review software [29], and additional duplicates were removed. Before screening the articles, three researchers (YO, JZ, and YF) met to discuss the procedure for title and abstract screening using 20 randomly selected papers. In the first phase of screening, two researchers (YO and JZ) independently assessed all study titles and abstracts against the eligibility criteria in Table 1. The agreement in the abstract and title screening between the two reviewers was 97.4% (Cohen’s Kappa = .725). Then, they (YO and JZ) read the remaining studies in full length. The agreement for full text screening was 91.9% (Cohen’s Kappa = .734). Discrepancies at each stage were resolved through discussion with a third researcher (YF).

### Data collection process and data items

Data extraction forms were developed based on the AI Chatbot Behavior Change Model [22], which provides a comprehensive framework for analyzing and evaluating chatbot designs and technologies. This model consists of four major components that provide guidelines to develop and evaluate AI chatbots for health behavior changes: 1) designing chatbot characteristics and understanding user background, 2) building relational capacity, 3) building persuasive capacity, and 4) evaluating mechanisms and outcomes. Based on the model, the data extraction forms were initially drafted by YF and discussed among the research team members. One researcher (YO) extracted information on study and sample characteristics, chatbot characteristics, intervention characteristics, outcome measures and results for main outcomes (i.e., PA, diet, and weight loss) and secondary outcomes (i.e., engagement, acceptability/satisfaction, adverse events, and others). Study and sample characteristics consisted of study aim, study design, theoretical framework, sample size, age, sex/gender, race/ethnicity, education, and income. Chatbot characteristics included the systematic features the chatbots were designed with (i.e., chatbot name and gender, media, user input, conversation initiation, relational capacity, persuasion capacity, safety, and ethics discussion). Intervention characteristics included information such as intervention duration and frequency, intervention components, and technological features (e.g., system infrastructure, platform). Two researchers (YF and JZ) independently validated the extracted data.

### Quality assessment and risk of bias

Two reviewers (YO and JZ) independently evaluated the risk of bias of included studies using the two National Institutes of Health (NIH) quality assessment tools [30]. Randomized controlled trials (RCTs) were assessed for methodological quality using the NIH Quality Assessment of Controlled Intervention Studies. For quasi-experimental studies, the NIH Quality Assessment Tool for Before-After (Pre-Post) Studies with No Control Group was used. Using these tools, the quality of each study was categorized into three groups (“good,” “fair,” and “poor”). These tools were used to assess confidence in the evaluations and conclusions of this systematic review. We did not use these tools to exclude the findings of poor quality studies. It should be noted that the studies included in this systematic review were behavioral intervention trials targeting individual-level outcomes. Therefore, criteria asking 1) whether participants did not know which treatment group they were assigned to and 2) the statistical analyses of group-level data were considered inapplicable.

### Synthesis of results

Due to the heterogeneity in the types of study outcomes, outcome measures, and clinical trial designs, we qualitatively evaluated and synthesized the results of the studies. We did not conduct a meta-analysis and did not assess publication bias.

## Results

### Study selection

**Fig. 1:**
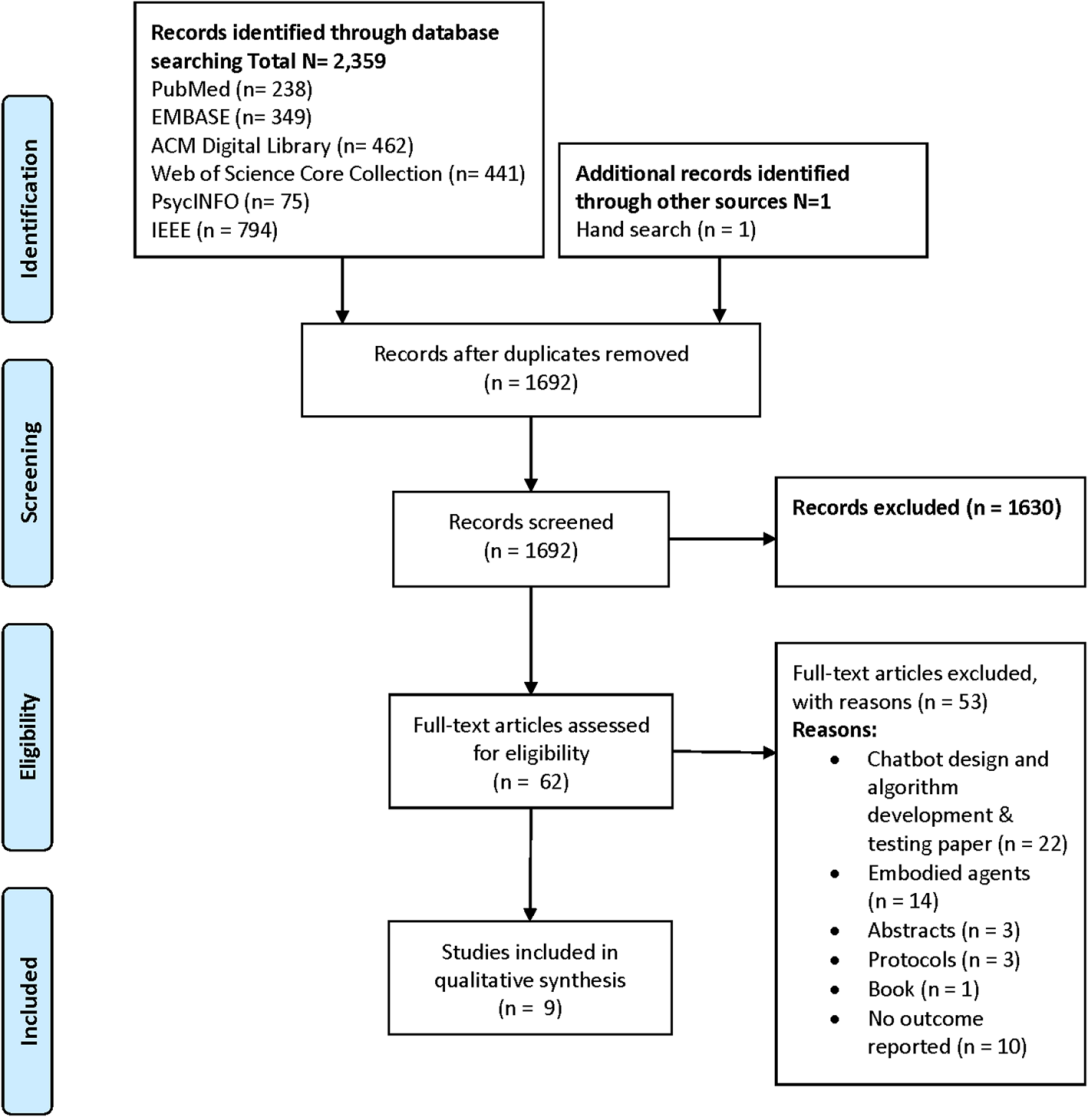
Flow diagram of the article screening process

Figure 1 shows the study selection process. The search yielded 2360 references in total, from which 668 duplicates were removed. A total of 1692 abstracts were then screened, among which 1630 were judged ineligible, leaving 62 papers to be read in full text. In total, 9 papers met the eligibility criteria and were included.

### Summary of study designs and sample characteristics

The 9 included papers had been recently published (3 were published in 2020 [20, 31, 32], 4 in 2019 [21, 33–35], and 2 in 2018 [36, 37]). Table 2 provides details of the characteristics of each study. Two studies [21, 37] were conducted in the United States and the remaining 7 were conducted in Switzerland [31, 33, 36], Australia [20], South Korea [32], and Italy [34] (1 not reported [35]). In total, 891 participants were represented in the 9 studies, with sample sizes ranging from 19 to 274 participants. The mean age of the samples ranged from 15.2 to 56.2 years (SD _range_ = 2.0 to 13.7), and females/women represented 42.1 to 87.9% of the sample. One study [21] solely targeted an adolescent population, whereas most studies targeted an adult population [20, 31–35, 37]. One study [36] did not report the target population’s age. Participants’ race/ethnicity information was not reported in 8 out of the 9 studies. The study [21] that reported participants’ race/ethnicity information included 43% Hispanic, 39% White, 9% Black, and 9% Asian participants. Participants’ education and income backgrounds were not reported in 5 out of the 9 studies. Among the 4 studies [31, 34, 35, 37] that reported the information, the majority included undergraduate students or people with graduate degrees. Overall, reporting of participants’ sociodemographic information was inconsistent and insufficient across the studies.

**Table 2:** Summary of study and sample characteristics

No.	First author/published year/Country	Primary Aim(s)	Study design/# of groups	Theoretical framework	Sample characteristics				
					Total Size (N)/Attrition (%)	Mean age (SD) years and/or range	Females/ Women %	Race/ Ethnicity %	Education/Income
**Randomized controlled trials**									
1	Kramer J/^a^ 2020/Switzerland [31]	To evaluate the effects of the Ally chatbot that combines financial incentives, weekly planning, and daily self-monitoring prompts on reaching daily step goals.	Optimization randomized trial/1 group micro-randomized to incentive (cash vs. charity vs. no incentive) X Planning (action vs. coping vs. no planning) X Self-monitoring prompt (prompt vs. no prompt) groups.	Health Action Process Approach	*N* = 274/30.3%	41.7 (13.5)/NR	57.7	NR	59.9% with university degree
2	Kunzler F/^b^2019/Switzerland [33]	To explore the factors affecting users’ receptivity towards Just-In-Time Adaptive Interventions (JITAIs) delivered via the Ally chatbot.	Randomized controlled trial/ 3 groups (cash bonus vs. charity donation vs. control)	NR	*N* = 189/NR	40.0 (13.7)/NR	63.0	NR	NR
3	Piao M/ 2020/ South Korea [32]	To assess the efficacy of the Healthy Lifestyle Coaching Chatbot intervention presented via a messenger app aimed at stair-climbing habit formation for office workers.	Randomized Controlled Trial/ 2 groups (intervention vs. control)	Habit Formation Model	*N* = 106/12.3%	NR/20-59	56.7	NR	NR
4	Carfora V/2019/Italy [34]	To test a chatbot that delivers daily messaging intervention aimed at promoting the reduction of red and processed meat consumption (RPMC).	Randomized Controlled Trial/3 groups (informational vs. emotional vs. control)	NR	*N* = 180/8.0%	20 (2.0)/NR	75.6	NR	100% Undergraduate students
**Non-randomized studies**									
5	Maher CA/2020/Australia [20]	To test the feasibility (recruitment and retention) and preliminary efficacy of physical activity and Mediterranean-style dietary intervention (MedLiPal) delivered via an artificially intelligent virtual health coach.	Quasi-experiment/1 group	NR	*N* = 31/9.7%	56.2 (8.0)/45-75	67.7	NR	NR
6	Fadhil A/2019/NR [35]	To present the design and validation of CoachAI, a conversational agent-assisted health coaching system on physical activity, healthy diet, and stress coping.	Quasi-experiment/1 group	Health Action Process Approach, Technology Acceptance Model, AttrakDiff Model	*N* = 19/NR	28.5 (9.4)/19-53	42.1	NR	Most were university students or had graduate degree
7	Stephens TN/2019/U.S. [21]	To assess the feasibility of integrating the Tess chatbot in behavioral counseling of adolescent patients coping with weight management and prediabetes symptoms to promote treatment adherence, behavior change, and overall wellness.	Quasi-experiment/1 group	Cognitive Behavioral Therapy, Emotionally Focused Therapy, Behavioral Activation, Motivational Interviewing	*N* = 23/NR	15.2 (NR)/9.78-18.54	57.0	Hispanic (43%), White (39%), Black (9%), Asian (9%)	NR
8	Casas J/ 2018/ Switzerland [36]	To evaluate the effects of a conversational assistant designed to monitor and coach participants to achieve specific goals regarding their diet.	Quasi-experiment/1 group	NR	*N* = 36/NR	NR	NR	NR	NR
9	Kocielnik R/ 2018/ U.S. [37]	To develop and examine the feasibility of a mobile conversational system, Reflection Companion, to engage users in reflection on physical activity through dialogues	Quasi-experiment/1 group	Structured Reflection Model	*N* = 33/NR	36.5 (11.2)/21-60	87.9	NR	55% college degree or being enrolled in college, 27% graduate degree

Studies a and b employed the same chatbot named Ally

*NR* not reported

Five studies employed quasi-experimental designs [20, 21, 35–37], and 4 were RCTs [31–34]. Only 5 studies [21, 31, 32, 35, 37] used at least one theoretical framework. One was guided by 3 theories [35] and another by 4 theories [21]. The theories used in the 5 studies included the Health Action Process Approach (*n* = 2), the Habit Formation Model (*n* = 1), the Technology Acceptance Model (*n* = 1), the AttrakDiff Model (*n* = 1), Cognitive Behavioral Therapy (*n* = 1), Emotionally Focused Therapy (*n* = 1), Behavioral Activation (*n* = 1), Motivational Interviewing (*n* = 1), and the Structured Reflection Model (*n* = 1). It is notable that most of these theories were used to design the intervention contents for inducing behavioral changes. Only the Technology Acceptance Model and the AttrakDiff Model were relevant for guiding the designs of the chatbot characteristics and their technological platforms, independent from intervention contents.

### Summary of intervention and chatbot characteristics

**Fig. 2:**
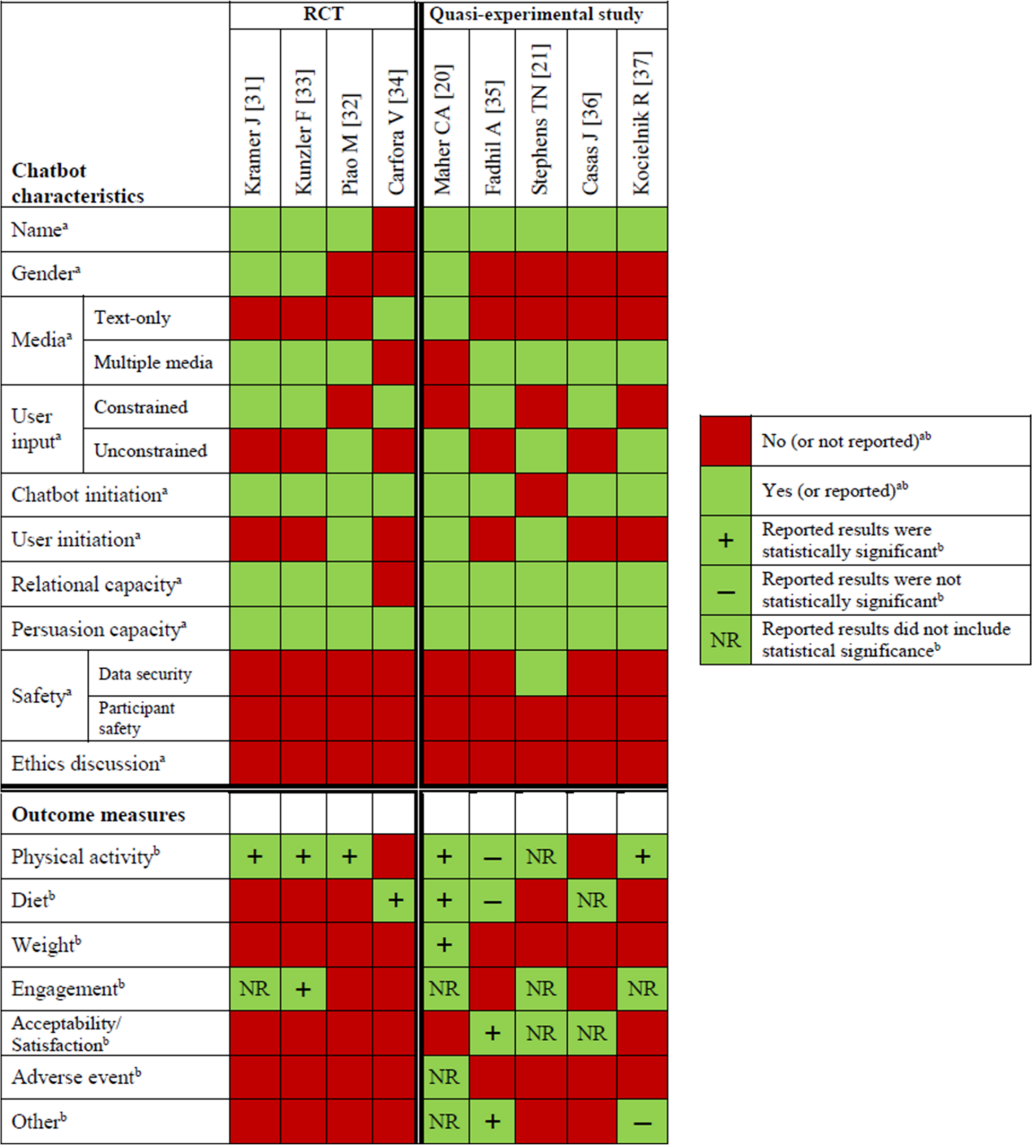
Summary of chatbot characteristics and intervention outcomes

Figure 2 provides a visual summary of AI chatbot characteristics and intervention outcomes, and Table 3 provides more detailed information. The 9 studies varied in intervention and program length, lasting from 1 week to 3 months. For most studies (*n* = 8), the chatbot was the only intervention component for delivering contents and engaging with the participants. One study used multi-intervention components, and participants had access to an AI chatbot along with a study website with educational materials [20]. A variety of commercially available technical platforms were used to host the chatbot and deliver the interventions, including Slack (*n* = 2), KakaoTalk (*n* = 1), Facebook messenger (*n* = 3), Telegram messenger (*n* = 1), WhatsApp (*n* = 1), and short messaging services (SMS) (*n* = 2). One study used 4 different platforms to deliver the intervention [21], and 2 studies used a chatbot app (i.e., Ally app) that was available on both Android and iOS systems [31, 33].

**Table 3:** Summary of chatbot and intervention characteristics

No.	First author/published year/Country	Intervention duration and frequency ^**1**^	Chatbot only or multiple components intervention ^**2**^	Overall technological features of the intervention ^**3**^	Chatbot characteristics							
					Chatbot identity (name, gender) ^**4**^	Media ^**5**^	User input ^**6**^	Chatbot initiation/ User initiation^**7**^	Relational capacity ^**8**^	Persuasion capacity ^**9**^	Safety ^**10**^	Ethics discussion ^**11**^
**Randomized controlled trials**												
1	Kramer J/^a^2020/Switzerland [31]	6 weeks and daily	Chatbot only	The Assistant to Lift your Level of activitY (Ally) app was developed using the MobileCoach platform, on both Android and iOS systems	Name: Ally Gender: Woman	Texts, graphs	Constrained	Daily and weekly messages delivered at a random time between 10 AM and 6 PM/NR	Using personalized greeting, initiating daily conversations, and occasionally sending unrelated messages to keep user interests	Setting personalized goals, using action-planning, coping-planning, and self-monitoring prompts	NR	NR
2	Kunzler F/^b^2019/Switzerland [33]	6 weeks and daily	Chatbot only	Ally app (available on both iOS and Android) is based on the MobileCoach platform. Physical activity was measured by subscribing to CoreMotion Activity Manager on iOS and Google Activity Recognition API	Name: Ally Gender: Woman	Texts, graphs	Constrained	Daily messages delivered 3 times a day (between 8 and 10 AM, 10-6 PM, or 8 PM) and weekly messages delivered at random times/NR	Initiating daily conversations with a greeting	Using persuasive prompts such as goal setting, self-monitoring, goal achievement, and weekly planning	NR	NR
3	Piao M/2020/ South Korea [32]	12 weeks and daily	Chatbot only	The Healthy Lifestyle Coaching Chatbot was developed using the Watson Conversational tool (IBM Corp) and was linked to the KakaoTalk Smart Chat application programming interface (API) through the RESTful API. It was deployed through KakaoTalk messenger app	Name: Chatbot Gender: NR	Texts, Images	Unconstrained	Daily message delivered at participants’ specified time/On-demand	Sending personalized goal-related messages based on their daily routines, send a compliment message providing positive feedback	Setting personalized goals, providing extrinsic (e.g., financial incentives) and intrinsic (e.g., pleasure, satisfaction) were used.	NR	NR
4	Carfora V/2019/Italy [34]	2 weeks and daily	Chatbot only	Chatbot was deployed through Facebook Messenger	Name: NR Gender: NR	Texts	Constrained	Daily messages delivered at 7:30 AM/ NR	NR	Informing participants about the health and environmental impact of excessive RPMC; Using persuasive message appeal (i.e., emotional appeal such as regret)	NR	NR
**Non-randomized studies**												
5	Maher CA/2020/Australia [20]	12 weeks and weekly	Multicomponent	The Paola chatbot was developed using the IBM Watson Virtual Assistant AI software and was hosted on the cloud-based instant messaging platform Slack. The program also used the MedLipal website and the Garmin Vivofit4 physical activity monitor	Name: Paola Gender: Woman	Texts	Unconstrained	Weekly check-in messages/On-demand	Referring to users by their first name and responding to questions at any time.	Assisting participants to set a personal daily step count goal based on age-based normative values + 2000 steps. This daily step goal was revisited and edited at each weekly check-in.	NR	NR
6	Fadhil A/2019/NR [35]	3 weeks and daily	Chatbot only	Chatbot used the coaching portal combined with the dialogue engine which consists of rails state machine, user clustering model and fitbit wearable data. Chatbot was deployed through Telegram Messenger	Name: CoachAI Gender: NR	Texts, Images	Constrained	Messages delivered recurrently (e.g., daily, weekly, on weekends, or weekdays) or at a scheduled date and time/ NR	Using greeting and some preliminary evaluation chat at the beginning of the interaction and assessing users’ comfort in discussing personal information with the agent.	Providing motivational messages consisted of positive reinforcement and a feedback on user’s overall adherence for the week to increase adherence	NR	NR
7	Stephens TN/ 2019/ U.S. [21]	10–12 weeks NR	Chatbot only	The Tess chatbot was deployed through multiple channels (i.e., SMS text message, Slack, WhatsApp or Facebook Messenger) and also was integrated with Google Home and Amazon Alexa	Name: Tess Gender: NR	Texts, Voice	Unconstrained	NR/On-demand	Mimicking empathy and compassion, adjusting the conversational style or modality to address each client’s needs, and responding based on the individual’s reported emotion or concern.	Specific goals and targeted behaviors were entered to the system to offer individualized conversations. Delivered customized integrative support, psychoeducation, and interventions	Data processing and storage are on secure servers that satisfy Health Insurance Portability and Accountability Act (HIPAA) regulations and within the country of residence for all participants given access.	NR
8	Casas J/2018/Switzerland [36]	Seven days and daily	Chatbot only	The Rupert chatbot was developed using the Chatfuel service, and deployed through Facebook Messenger	Name: Rupert le nutritionniste Gender: NR	Texts, graphs, images, videos	Constrained	Daily messages/NR	Showing empathy, being friendly, positive, and not judgmental; speaking in users’ native language (i.e., French)	Asking participants to choose their goals, monitoring food intake, answering questions, and giving recommendations	NR	NR
9	Kocielnik R/ 2018/ U.S. [37]	2 weeks and daily	Chatbot only	Twillio API was used to communicate with users via mobile phones through SMS/MMS. Fitbit API was queried for user activity data to generate activity graphs. LUIS API offered automated recognition of free-text user responses	Name: Reflection Companion Gender: NR	Texts, Graphs	Unconstrained	Daily messages/NR	Personalized the experience by introducing questions that referenced users’ own behavior change goals	The mini-dialogues were delivered with a graph showing user’s physical activity metrics	NR	NR

Studies a and b employed the same chatbot named Ally.

*NR* not reported.

^1^ Intervention duration is how long the intervention lasted and frequency is how often the programed intervened with the participants

^2^ Multicomponent means the intervention had multiple intervention components (e.g., in-person and using chatbots); chatbot only means the intervention was sorely delivered by the chatbot

^3^ Document the technological infrastructure, platform, and features of the intervention

^4^ Chatbot identity documents identity cues the chatbot is designed with. The cues can include name, gender, age, etc.

^5^ Media documents the types of media that the chatbot can use to deliver information

^6^ User inputs document the capacity of which participants can interact with the chatbot. Constrained means users can only select pre-programmed responses in the chat; unconstrained means users can freely type or speak to the chatbot

^7^ Chatbot/User initiation indicates whether and how often chatbot/user initiated the conversation

^8^ Relational capacity documents conversation strategies the chatbot can use to establish, maintain, or enhance social relationships with the participants (e.g., greetings)

^9^ Persuasion capacity documents conversation strategies the chatbot can use to change participant’s behaviors and behavioral determinants (e.g., knowledge, attitudes, norm perceptions, efficacy, etc.)

^10^ Safety documents strategies the chatbot is designed to ensure safety of the participants

^11^ Ethics discussion documents any ethical principles or standards the chatbot is designed with. Key ethical considerations include having transparency and user trust, protecting user privacy, and minimizing biases

Following the AI Chatbot Behavior Change Model [22], we extracted features of the chatbot and intervention characteristics (Table 3). Regarding chatbot characteristics, identity features, such as specific names (*n* = 8) [20, 21, 31–33, 35–37] and chatbot gender (*n* = 3) [20, 31, 33], were specified. Notably, the chatbot gender was woman in the 3 studies that reported it [20, 31, 33]. All 9 chatbots delivered messages in text format. In addition to text, 3 chatbots used graphs [31, 33, 37], 2 used images [32, 35], 1 used voice [21], and 1 used a combination of graphs, images, and videos [36].

In 5 studies, the chatbots were constrained (i.e., users could only select pre-programmed responses in the chat) [31, 33–36], and in 4, the chatbots were unconstrained (i.e., users could freely type or speak to the chatbot) [20, 21, 32, 37]. Six chatbots [31–34, 36, 37] delivered daily intervention messages to the study participants. One chatbot communicated only on a weekly basis [20], and 1 communicated daily, weekly, on weekends or weekdays or at a scheduled date and time [35]. One study did not specify when and how often the messages were delivered [21]. Only 3 chatbots [20, 21, 32] were available on-demand so that users could initiate conversation at any preferred time. Most chatbots were equipped with relational capacity (*n* = 8; i.e., conversation strategy to establish, maintain, or enhance social relationships with users) and persuasive capacity (*n* = 9; i.e., conversation strategy to change user’s behaviors and behavioral determinants), meaning that the conversations were designed to induce behavioral changes while engaging with users socially. While only 1 study [21] documented data security, none of the studies provided information on participant safety or ethics (i.e., ethical principle or standards with which the chatbot is designed).

### Summary of outcome measures and changes in outcomes

Figure 2 also illustrates the outcome measures and changes in the main and secondary outcomes reported in both RCTs and quasi-experimental studies. Among 7 studies that measured PA [20, 21, 31–33, 35, 37], 2 used objective measures [31, 33], 4 used self-reported measures [20, 21, 32, 35], and 1 used both [37]. Self-reported dietary intake was measured in 4 studies [20, 34–36]. Only 1 study assessed objective changes in weight in a research office visit [20]. Details of intervention outcomes, including direction of effects, statistical significance, and magnitude, are presented in Table 4.

**Table 4:** Summary of outcome measures and results

No.	First author/published year/Country	Main outcome measures			Secondary outcome measures			
		Physical activity (PA)	Diet	Weight	Engagement	Acceptability and satisfaction	Adverse event	Other outcomes
		Results	Results	Results	Results	Results	Results	Results
**Randomized controlled trials**								
1	Kramer J/^a^ 2020/ Switzerland [31]	OM (Daily step count obtained from smartphone)	NR	NR	OM (Rate of individuals who stopped using the app)	NR	NR	NR
		Daily cash incentives increased step-goal achievement by 8.1% (CI: [2.1, 14.1]) and, only in the no-incentive control group, action planning increased step-goal achievement by 5.8% (CI: [1.2, 10.4]).	NR	NR	30% of participants stopped using the app over the course of the study.	NR	NR	NR
2	Kunzler F/^b^ 2019/ Switzerland [33]	OM (Daily step count obtained from smartphone)	NR	NR	OM (Just-in-time-response rate, overall response rate, conversation engagement, response delay obtained from the chatbot)	NR	NR	NR
		Physical activity goal completion rate was correlated with overall response rate (*r* = 0.53, *p* < 0.001), just-in-time response rate (*r* = 0.42, *p* < 0.001), conversation rate (r = 0.38, *p* < 0.001) and average response delay (*r* = − 0.27, *p* < 0.001).	NR	NR	Intrinsic factors: Device type, age, and personality traits had a significant effect on the just-in-time response rate, conversation rate, and total response rate. Extrinsic factors: Time and day of the delivery, phone battery, device interaction, and location had significant effects on just-in-time response, conversation engagement, and response delay.	NR	NR	NR
3	Piao M/ 2020/ South Korea [32]	SR (Self-Report Habit Index)	NR	NR	NR	NR	NR	NR
		After 4 weeks of intervention without providing the intrinsic rewards in the control group, the change in SRHI scores was 13.54 (SD ± 14.99) in the intervention group and 6.42 (SD ± 9.42) in the control group (*p* = .04). When all rewards were given to both groups, from the fifth to twelfth week, the change in SRHI scores of the intervention and control groups was comparable at 12.08 (SD ± 10.87) and 15.88 (SD ± 13.29), respectively (*p* = .21) The level of physical activity showed a significant difference between the groups after 12 weeks of intervention (*p* = .045)	NR	NR	NR	NR	NR	NR
4	Carfora V/ 2019/ Italy [34]	NR	SR (Self-reported RPMC; intention; attitude; regret on RPMC)	NR	NR	NR	NR	NR
		NR	The emotional condition had stronger anticipated regret and higher intention to reduce RPMC, as compared to the control condition (*p* = .01 and *p* = .02 respectively). Both emotional and informational groups showed lower self-reported RPMC as compared to control. (*p* = .03 and *p* = .05 respectively).	NR	NR	NR	NR	NR
**Non-randomized studies**								
5	Maher CA/ 2020/ Australia [20]	SR (Active Australia Survey)	SR (14-item Australian Mediterranean Diet Adherence)	OM (Seca 703)	OM (Number of weekly check-in obtained from the chatbot)	NR	NR	OM (Feasibility of subject enrollment)
		Increased MVPA 109.8 (95% CI 1.9 to 217.7, *p* = .005) minutes per day from baseline to 12 weeks.	Increased 5.7 (95% CI 4.2 to 7.3, *p* < .001) points in diet adherence from baseline to 12 weeks.	Lost 1.3 (95% CI − 25 to − 0.7, *p* = .01) kg from baseline to 12 weeks.	Mean weekly chatbot interaction 6.9 times out of 11 possible interactions.	NR	No adverse events reported	Enrolled 31 out of 99 screened participants in the 6- week enrollment period
6	Fadhil A/2019/NR [35]	SR (Physical activity intention)	SR (Healthy diet intention)	NR	NR	SR (TAM questionnaire)	NR	SR (AttrakDiff questionnaire)
		Results showed no difference between the three weeks; the scores remained unchanged for the physical activity.	Results showed no difference between the three weeks; the scores remained unchanged for the healthy diet.	NR	NR	The scales “ease of use,” “attitude,” and “intention” towards using the system were significantly higher than the middle score (respectively: *t*(17) = 4.9, *p* < .01; *t*(17) = 2.5, *p* < .05; *t*(17) = 3.1, *p* < .01).	NR	Average scores were statistically higher than 4 for each dimension: pragmatic (*t*(17) = 5.41, *p* < .01), hedonic (*t*(17) = 3.4, *p* < .01), appealing (*t*(17) = 4.2, *p* < .01), and social (*t*(17) = 2.6, *p* < .05).
7	Stephens/2019/ U.S. [21]	SR (Target goal progress)	NR	NR	OM (Duration of conversation, Quantity of messages exchanged, Number of hours support exchanged, Percentage of exchanges outside of typical office hours obtained from the chatbot, Ratio of chatbot-initiated vs. patient-initiated conversations obtained from the chatbot)	SR (Helpfulness)	NR	NR
		Adolescent patients reported experiencing positive progress toward their goals 81% of the time.	NR	NR	A total of 4123 messages were exchanged between participants and Tess. The average duration of conversations between Tess and patients was approximately 12.5 min (*SD* = ± 15.62 min). The median length of conversations was nearly 6 min, Tess provided about 55 h and 45 min of support for the adolescent patients, 17.8% of which was provided outside of typical office hours. A majority of the conversations were Tess initiated (73.6%) compared to patient initiated.	Patients indicated that Tess was helpful 96% of the time.	NR	NR
8	Casas J/2018/ Switzerland [36]	NR	SR (Meal consumption)	NR	NR	SR (Chatbot Effectiveness)	NR	NR
		NR	Only 11% of participants succeeded with their goals. In 65% of the cases the person has improved his consumption. In 12% of cases, consumption remained stable and in the remaining 24%, their consumption has worsened.	NR	NR	82% of participants said that Rupert allowed them to think and be aware of their consumption. 86% reported answering honestly to the daily requests of the chatbot. 70% thought the chatbot intervention was efficient.	NR	NR
9	Kocielnik R/ 2018/ U.S. [37]	SR (Habituation Action; Understanding; Reflection; Critical reflection adapted from Kember et al. 2000) OM (Step count obtained from fitness trackers) SR (Physical activity awareness)	NR	NR	OM (Participant interactions with the system: 1) number of dialogues responded to, 2) the time until a response was made, 3) the length and content of responses obtained from the chatbot) SR (Willingness to use the system for additional 2 weeks without compensation)	NR	NR	SR (Mindfulness)
		Significant difference in Habitual Action (HA) for pre (M = 3.16, SD = 1.06) to post (M = 3.53, SD = 0.89) study measurements; *t*(32) = −2.04, *p* < 0.05. A weakly significant increase in Understanding (U) from pre (M = 3.60, SD = 0.98) to post (M = 3.92, SD = 0.84); *t*(32) = −1.90, *p* = 0.07. Step count difference was not significant. Physical activity awareness difference was not significant	NR	NR	Participants responded to 96% of all initial questions and to 90% of the follow-up questions sent by the system. 16 out of the 33 participants elected to continue using the system for 2 additional weeks without reward.	NR	NR	No significant changes were observed between pre- and post measurements

Studies a and b employed the same chatbot named Ally

*PA* physical activity, *SR* self-report, *OM* objective measure, *MVPA* moderate to vigorous physical activity, *RPMC* red and processed meat consumption, *NR* not reported

Sample sizes of the 4 RCT studies ranged from 106 to 274 and a priori power analyses were reported in 3 [31, 32, 34], which showed that the sample sizes had sufficient power for analyzing the specified outcomes. Of the 4 RCT studies [31–34], 3 reported PA outcomes using daily step count [31, 33] and a self-reported habit index [32]. In these RCTs, the AI chatbot intervention group resulted in a significant increase in PA, as compared to the control group, over the respective study period (6 weeks to 3 months). In terms of dietary change, 1 study [34] reported that participants in the intervention group showed higher self-reported intention to reduce red and processed meat consumption compared to the control group during a 2-week period.

In contrast, sample sizes for the 5 quasi-experimental studies were small, ranging from 19 to 36 participants, suggesting that these studies may lack statistical power to detect potential intervention effects. Among the 5 quasi-experimental studies, 2 [21, 37] reported only PA change outcomes, 1 [36] reported only diet change outcomes, and 2 [20, 35] reported both outcomes. With regard to PA-related outcomes, 2 studies reported statistically significant improvements [20, 37]. Specifically, [20] observed increased moderate and vigorous PA over the study period [37]. found a significant increase in the habitual action of PA. One study [35] found no difference in PA intention within the intervention period. Although this study did not observe a statistically significant increase in PA intention, it revealed that among participants with either high or low intervention adherence, their PA intention showed an increasing trend over the study period [21]. only reported descriptive statistics and showed that participants experienced positive progress towards PA goals 81% of the time.

Among the quasi-experimental studies, only 1 study reported a statistically significant increase in diet adherence over 12 weeks [20] [35]. reported no difference of healthy diet intention over 3 weeks. In this study, participants with high intervention adherence showed a marginal increase, whereas, those with low adherence showed decreased healthy diet intention [36]. reported that participants’ meal consumption improved in 65% of the cases. The only study [20] reporting pre-post weight change outcomes using objective weight measures showed that participants experienced a significant weight loss (1.3 kg) from baseline to 12 weeks. To summarize, non-significant findings and a lack of statistical reporting were more prevalent in the quasi-experimental studies, but the direction of intervention effects were similar to those reported in the RCTs.

Engagement, acceptability/satisfaction, and safety measures were reported as secondary outcomes in 7 studies [20, 21, 31, 33, 35–37]. Five studies reported engagement [20, 21, 31, 33, 37] using various types of measurements, such as user response rate to chatbot messages [31], frequency of users’ weekly check-ins [20], and length of conversations between the chatbot and users [21]. Three studies measured acceptability/satisfaction of the chatbot [21, 35, 36] using measures such as technology acceptance [35], helpfulness of the chatbot [21], and perceived efficiency of chatbot communications [36]. Regarding reporting of adverse events (e.g., experiencing side effects from interventions), only 1 study reported that no adverse events related to study participation were experienced [20]. Three studies reported additional measures, including feasibility of subject enrollment [20], using the AttrakDiff questionnaire for measuring four aspects of the chatbot (i.e., pragmatic, hedonic, appealing, social) [35], and assessing perceived mindfulness about own behaviors [37].

Among 5 studies that reported engagement [20, 21, 31, 33, 37], only 1 [33] reported statistical significance of the effects of intrinsic (e.g., age, personality traits) and extrinsic factors (e.g., time and day of the delivery, location) on user engagement (e.g., conversation engagement, response delay). Among 3 studies [21, 35, 36] that reported acceptability/satisfaction, 1 study [35] found that the acceptability of the chatbot was significantly higher than the middle score corresponding to “neutral” (i.e., 4 on a 7-point scale). One study that reported the safety of the intervention did not include statistical significance [20]. Three studies reported other measures [20, 35, 37], and 1 found that pragmatic, hedonic, appealing, and social ratings of the chatbot were significantly higher than the middle score [35]. Another study [37] found no significant changes in the perceived mindfulness between pre- and post-study.

### Summary of quality assessment and risk of bias

The results of risk of bias assessments of the 9 studies are reported in Additional file 2. Of the 4 RCT studies [31–34], 3 were rated as fair [31, 32, 34] and 1 was rated as poor [33] due to its lack of reporting of several critical. The poorly rated study did not report overall dropout rates or the differential dropout rates between treatment groups, did not report that the sample size was sufficiently large to be able to detect differences between groups (i.e., no power analysis), and did not prespecify outcomes for hypothesis testing. Of the 5 quasi-experimental studies [20, 21, 35–37], 1 study was rated as fair [20] and 4 studies were rated as poor [21, 35–37] due to flaws with regard to several critical. These studies reported neither a power analysis to ensure that the sample size was sufficiently large, nor follow-up rates after baseline. Additionally, the statistical methods did not examine pre-to-post changes in outcome measures and lacked reporting of statistical significance.

## Discussion

This systematic review aimed to evaluate the characteristics and potential efficacy of AI chatbot interventions to promote PA, healthy diet, and/or weight management. Most studies focused on changes in PA, and majority [20, 31–33, 37] reported significant improvements in PA-related behaviors. The number of studies with the aim to change diet and weight status was small. Two studies [20, 34] found significant improvements in diet-related behaviors. Although only 1 study [20] reported weight-related outcomes, it reported significant weight change after the intervention. In summation, chatbots can improve PA, but the study not able to make definitive conclusions on the potential efficacy of chatbot interventions on promoting PA, healthy eating, or weight loss.

This qualitative synthesis of effects needs to be interpreted with caution given that the reviewed studies lack consistent usage of measurements and reporting of outcome evaluations. These studies used different measurements and statistical methods to evaluate PA and diet outcomes. For example, 1 study [20] measured one’s self-reported change in MVPA during the intervention period to gauge the efficacy of the intervention, whereas in another study [31] step-goal achievement was used as a measure of the intervention efficacy. The two quasi-experimental studies did not report statistical significance of the pre-post changes in PA or diet outcomes [21, 36]. Such inconsistency in evaluating the potential efficacy of interventions has been reported in previous systematic reviews [1, 38]. To advance the application of chatbot interventions in lifestyle modification programs and to demonstrate the rigor of their efficacy, future studies should examine multiple behavior change indicators, ideally incorporating objectively measured outcomes.

Consistent with other systematic reviews of chatbot interventions in health care and mental health [1, 38], reporting of participants’ engagement, acceptability/satisfaction, and adverse events was limited in the studies. In particular, engagement, acceptability, and satisfaction measures varied across the studies, impeding the systematic summarization and assessment of various intervention implementations. For instance, 1 study [33] used user response rates and user response delay as engagement measures, whereas in another study [21], the duration of conversation and the ratio of chatbot-initiated on patient-initiated conversations were used to assess the level of user engagement. Inconsistent reporting of user engagement, acceptability, and satisfaction measures may be problematic because it could contribute challenges to the interpretation and comparison of the results across different chatbot systems [1]. Therefore, standardization of these measures should be implemented in future research. For example, as suggested in previous studies [39, 40], conversational turns per session can be a viable, objective, and quantitative metric for user engagement. Regarding reporting of adverse events, despite the recommendation of reporting adverse events in clinical trials by the Consolidated Standards of Reporting Trials Group [41], only 1 study [20] reported adverse events. It is recommended that future studies consistently assess and report any unexpected events resulting from the use of AI chatbots to prevent any side effects or potential harm to participants.

Theoretical frameworks for designing and evaluating a chatbot system are essential to understand the rationale behind participants’ motivation, engagement, and behaviors. However, theoretical frameworks were not reported in many of the studies included in this systematic review. The lack of theoretical foundations of existing chatbot systems has also been noted in previous literature [42]. In this review, we found that the majority of AI chatbots were equipped with persuasion strategies (e.g., setting personalized goals) and relational strategies (e.g., showing empathy) to establish, maintain, or enhance social relationships with participants. The application of theoretical frameworks will guide in developing effective communicative strategies that can be implemented into chatbot designs. For example, designing chatbots with personalized messages can be more effective than non-tailored and standardized messages [43, 44]. For relational strategies, future studies can benefit from drawing on the literature on human-computer interaction and relational agents (e.g., [45, 46]) and interpersonal communication theories (e.g., Social Penetration Theory [47]) to develop strategies to facilitate relation formation between participants and chatbots.

Regarding designs of chatbot characteristics and dialogue systems, the rationale behind using human-like identity features (e.g., gender selection) on chatbots was rarely discussed. Only 1 study [31] referred to literature on human-computer interaction [48] and discussed the importance of using human-like identity features on chatbots to facilitate successful human-chatbot relationships. Additionally, only one chatbot [21] was able to deliver spoken outputs. This is inconsistent with a previous systematic review on chatbots used in health care, in which spoken chatbot output was identified as the most common delivery mode across the studies [1].

With regard to user input, over half of the studies [31, 33–36] used a constrained AI chatbot, while the remaining [20, 21, 32, 37] used unconstrained AI chatbots. Constrained AI chatbots are rule-based, well-structured, and easy to build, control, and implement, thus ensuring the quality and consistency in the structure and delivery of content [42]. However, they are not able to adapt to participants’ inquiries and address emergent questions, and are, thus, not suitable for sustaining more natural and complex interactions with participants [42]. In contrast, unconstrained AI chatbots are known to simulate naturalistic human-to-human communication and may strengthen interventions in general, particularly in the long-term, due to their flexibility and adaptability in conversations [1, 38, 42]. With increasing access to large health care datasets, advanced technologies [49], and new developments in machine learning that allow for complex dialogue management methods and conversational flexibility [1], employing unconstrained chatbots to yield long-term efficacy may become more feasible in future research. For instance, increasing the precision of natural language understanding and generation will allow for AI chatbots to better engage users in conversations and follow up with tailored intervention messages.

Safety and data security criteria are essential in designing chatbots. However, only 1 study provided descriptions of these criteria. Conversations between study participants and chatbots should be carefully monitored since erroneous chatbot responses may result in unintended harm. In particular, as conversational flexibility increases, there may be an increase in potential errors associated with natural language understanding or response generation [1]. Thus, using unconstrained chatbots should be accompanied with careful monitoring of participant and chatbot interactions, and of safety functions.

## Strengths and limitations

This review has several strengths. First, to the best of our knowledge, this is the first review to systematically examine the characteristics and potential efficacy of AI chatbot interventions in lifestyle modifications, thereby providing crucial insights for identifying gaps and future directions for research and clinical practice. Second, we developed comprehensive search strategies with an MLS for six electronic databases to increase the sensitivity and comprehensiveness of our search. Despite its strengths, several limitations need to also be acknowledged. First, we did not search gray literature in this systematic review. Second, we limited our search to peer-reviewed studies published as full-text in English only. Lastly, due to the heterogeneity of outcome measures and the limited number of RCT designs in this systematic review, we were not able to conduct a meta-analysis and make firm conclusions of the potential efficacy of chatbot interventions. In addition, the small sample sizes used by the studies made it difficult to scale the results to general populations. More RCTs with larger sample sizes and longer study durations are needed to determine the efficacy of AI chatbot interventions on improving PA, diet, and weight loss.

## Conclusions

AI chatbot technologies and their commercial applications continue to rapidly develop, as do the number of studies about these technologies. Chatbots may improve PA, but this study was not able to make definitive conclusions of the potential efficacy of chatbot interventions on PA, diet, and weight management/loss. Despite the rapid increase in publications about chatbot designs and interventions, standard measures for evaluating chatbot interventions and theory-guided chatbots are still lacking. Thus, there is a need for future studies to use standardized criteria for evaluating chatbot implementation and efficacy. Additionally, theoretical frameworks that can capture the unique factors of human-chatbot interactions for behavior changes need to be developed and used to guide future AI chatbot interventions. Lastly, as increased adoption of chatbots will be expected for diverse populations, future research needs to consider equity and equality in designing and implementing chatbot interventions. For target populations with different sociodemographic backgrounds (e.g., living environment, race/ethnicity, cultural backgrounds, etc.), specifically tailored designs and sub-group evaluations need to be employed to ensure adequate delivery and optimal intervention impact.

## Supplementary Information

**Additional file 1.** Search strategies for PubMed, EMBASE, ACM Digital Library, Web of Science, PsycINFO, and IEEE.

**Additional file 2.** Summary of quality assessment and risk of bias.

## Data Availability

Not applicable.

## Abbreviations

AI: Artificial Intelligence
PROSPERO: International Prospective Register of Systematic Reviews
RCT: Randomized controlled trial
PA: Physical activity
MLS: Medical librarian
