# Supplementary material for: A systematic review of artificial intelligence chatbots for promoting physical activity, healthy diet, and weight loss
Source: Int J Behav Nutr Phys Act. 2021 Dec 11;18:160. doi: 10.1186/s12966-021-01224-6 (PMC8665320; doi:10.1186/s12966-021-01224-6)
Supplement: Supplementary file 1 — Additional file 1. Search strategies for PubMed, EMBASE, ACM Digital Library, Web of Science, PsycINFO, and IEEE. [file 12966_2021_1224_MOESM1_ESM.docx]

### Additional file 1. Search strategies for PubMed, EMBASE, ACM Digital Library, Web of Science, PsycINFO, and IEEE

**PubMed**

| # | Searches |
| --- | --- |
| 1 | ("Ai agent" OR “Ai agents” OR "Artificial agent" OR "Artificial agents" OR Artificial intelligence chatbot OR Artificial intelligence chatbots OR "Assistance technology" OR "Assistance technologies" OR “Chat bot”  OR “Chat bots” OR Chatbot OR Chatbots OR Chatterbot OR "Conversational agent" OR "Conversational agents" OR “Conversational AI” OR "Conversational assistant" OR "Conversational assistants" OR Conversational bot OR Conversational bots OR "Conversational interface" OR "Conversational interfaces" OR "Conversational system" OR "Conversational systems" OR "Dialog system" OR "Dialog systems" OR Dialogue agent OR "Dialogue system" OR "Dialogue systems"  OR "Digital assistant" OR "Digital assistants" OR Infobot OR "Intelligent agent" OR "Intelligent agents" OR Intelligent conversational assistant OR “Interactive agent” OR “Interactive agents” OR "Relational agent" OR "Relational agents" OR “Social robot” OR “Social robots” OR Virtual advisors OR "Virtual agent" OR "Virtual agents" OR “Virtual assistant” OR “Virtual assistants” OR “Virtual avatar” OR “Virtual avatars” OR “Virtual coach” OR “Virtual coaches” OR Virtual conversational agents) |
| 2 | (Diet [mh] OR Diet [tiab] OR Dietary [tiab] OR Exercise [mh] OR Exercise [tiab] OR Lifestyle [mh] OR Lifestyle [tiab] OR “life style” [tiab] OR “life styles” [tiab] OR Nutrition [mh] OR nutrition [tiab] OR “Physical activity” [tiab] OR “Physical activities” [tiab] OR “Sedentary behavior”[mh] OR “Sedentary behavior” [tiab] OR “Sedentary time” [tiab] OR sitting [tiab] OR “weight control” [tiab] OR “Weight loss” [mh] OR “Weight loss” [tiab] OR “Weight losses” [tiab] OR “Weight maintenance” [tiab]) |
| 3 | #1 AND #2  Limited by English |

**EMBASE**

| # | Searches |
| --- | --- |
| 1 | 'ai agent' OR 'ai agents' OR 'artificial agent' OR 'artificial agents' OR 'artificial intelligence chatbot' OR 'artificial intelligence chatbots' OR 'assistance technology' OR 'assistance technologies' OR 'chat bot' OR 'chat bots' OR chatbot OR chatbots OR chatterbot OR 'conversational agent' OR 'conversational agents' OR 'conversational ai' OR 'conversational assistant' OR 'conversational assistants' OR 'conversational bot' OR 'conversational bots' OR 'conversational interface' OR 'conversational interfaces' OR 'conversational system' OR 'conversational systems' OR 'dialog system' OR 'dialog systems' OR 'dialogue agent' OR 'dialogue system' OR 'dialogue systems' OR 'digital assistant' OR 'digital assistants' OR infobot OR 'intelligent agent' OR 'intelligent agents' OR 'intelligent conversational assistant' OR 'interactive agent' OR 'interactive agents' OR 'relational agent' OR 'relational agents' OR 'social robot' OR 'social robots' OR 'virtual advisors' OR 'virtual agent' OR 'virtual agents' OR 'virtual assistant' OR 'virtual assistants' OR 'virtual avatar' OR 'virtual avatars' OR 'virtual coach' OR 'virtual coaches' OR 'virtual conversational agents' |
| 2 | ‘body weight control’/exp OR ‘body weight control’ OR 'body weight loss'/exp OR 'body weight loss' OR ‘diet’/exp OR ‘diet’ OR dietary OR ‘nutrition’/exp OR ‘nutrition’ OR ‘physical activity, capacity and performance’/exp OR ‘physical activity’/exp OR ‘physical activity’ OR ‘physical activities’ OR ‘exercise’/exp OR ‘exercise’ OR 'lifestyle'/exp OR 'lifestyle' OR 'lifestyle modification'/exp OR 'lifestyle modification' OR 'life style'/exp OR 'life style' OR lifestyle OR lifestyles OR 'sedentary time'/exp OR 'sedentary time' OR 'sedentary lifestyle'/exp OR 'sedentary lifestyle' OR 'sitting'/exp OR 'sitting' |
| 3 | #1 AND #2  Limited by English  Limited by article and article in press |

**ACM Digital Library (The ACM Guide to Computing Literature)**

| # | **Search by title, keyword and abstract** |
| --- | --- |
| 1 | "ai agent" OR  "ai agents" OR  "artificial agent" OR  "artificial agents" OR  "artificial intelligence chatbot" OR  "artificial intelligence chatbots" OR  "assistance technology" OR  "assistance technologies" OR  "chat bot" OR  "chat bots" OR  chatbot OR  chatbots OR  chatterbot OR  "conversational agent" OR  "conversational agents" OR  "conversational ai" OR  "conversational assistant" OR  "conversational assistants" OR  "conversational bot" OR  "conversational bots" OR  "conversational interface" OR  "conversational interfaces" OR  "conversational system" OR  "conversational systems" OR  "dialog system" OR  "dialog systems" OR  "dialogue agent" OR “dialogue agents” OR   "dialogue system" OR  "dialogue systems" OR  "digital assistant" OR  "digital assistants" OR  infobot OR  "intelligent agent" OR  "intelligent agents" OR  "intelligent conversational assistant" OR  "interactive agent" OR  "interactive agents" OR  "relational agent" OR  "relational agents" OR  "social robot" OR  "social robots" OR  "virtual advisors" OR  "virtual agent" OR  "virtual agents" OR  "virtual assistant" OR  "virtual assistants" OR  "virtual avatar" OR  "virtual avatars" OR  "virtual coach" OR  "virtual coaches" OR  "virtual conversational agents" |
| 2 | (diet OR  dietary OR  exercise OR exercises OR “healthy eating” OR lifestyle OR lifestyles OR “life style” OR “life styles” OR nutrition OR  "physical activity" OR  "physical activities" OR  "sedentary behavior" OR  "sedentary time" OR  sitting OR "weight loss" OR  "weight losses" OR  "weight maintenance" OR “weight control”) |
| 3 | #1 AND #2 search by title  #1 AND #2 search by keyword  #1 AND #2 search by abstract |

**Web of Science Core Collection**

| # | **Topic Searches** |
| --- | --- |
| 1 | ("Ai agent" OR “Ai agents” OR "Artificial agent" OR "Artificial agents" OR “Artificial intelligence chatbot” OR “Artificial intelligence chatbots” OR "Assistance technology" OR "Assistance technologies" OR “Chat bot”  OR “Chat bots” OR Chatbot OR Chatbots OR Chatterbot OR "Conversational agent" OR "Conversational agents" OR “Conversational AI” OR "Conversational assistant" OR "Conversational assistants" OR “Conversational bot” OR “Conversational bots” OR "Conversational interface" OR "Conversational interfaces" OR "Conversational system" OR "Conversational systems" OR "Dialog system" OR "Dialog systems" OR “Dialogue agent” OR “Dialogue agents” OR "Dialogue system" OR "Dialogue systems"  OR "Digital assistant" OR "Digital assistants" OR Infobot OR "Intelligent agent" OR "Intelligent agents" OR “Intelligent conversational assistant” OR “Interactive agent” OR “Interactive agents” OR "Relational agent" OR "Relational agents" OR “Social robot” OR “Social robots” OR “Virtual advisors” OR "Virtual agent" OR "Virtual agents" OR “Virtual assistant” OR “Virtual assistants” OR “Virtual avatar” OR “Virtual avatars” OR “Virtual coach” OR “Virtual coaches” OR “Virtual conversational agents”) |
| 2 | (Diet OR Dietary OR Exercise OR Exercises OR Lifestyle OR Lifestyles OR” Life style” OR “Life styles” OR Nutrition OR "Physical activity" OR “Physical activities” OR “Sedentary behavior” OR “Sedentary time” OR “weight control” OR “Weight loss” OR “Weight losses” OR “Weight maintenance”) |
| 3 | #1 AND #2  Limited by article  Limited by Proceedings paper |

**PsycINFO**

| # | Searches |
| --- | --- |
| 1 | "Ai agent" OR “Ai agents” OR "Artificial agent" OR "Artificial agents" OR “Artificial intelligence chatbots” OR "Assistance technology" OR "Assistance technologies" OR “Chat bot” OR “Chat bots” OR IF (Chatbot) OR Chatbots OR Chatterbot OR MAINSUBJECT.EXACT("Conversational Agents") OR "Conversational AI” OR "Conversational assistant" OR "Conversational assistants" OR “Conversational bot” OR “Conversational bots” OR "Conversational interface" OR “Conversational interfaces" OR "Conversational system" OR "Conversational systems" OR "Dialog system" OR "Dialog systems" OR “Dialogue agent” OR "Dialogue system" OR "Dialogue systems" OR "Digital assistant" OR "Digital assistants" OR MAINSUBJECT.EXACT("Human Robot Interaction") OR Infobot OR MAINSUBJECT.EXACT("Intelligent Agents") OR “Intelligent conversational assistant” OR “Interactive agent” OR “Interactive agents” OR "Relational agent” OR "Relational agents" OR MAINSUBJECT.EXACT("Social Robotics") OR “Social robots” OR “Virtual advisors” OR "Virtual agent" OR "Virtual agents" OR “Virtual assistant” OR “Virtual assistants” OR “Virtual avatar” OR “Virtual avatars” OR “Virtual coach” OR “Virtual coaches” OR “Virtual conversational agents” |
| 2 | MAINSUBJECT.EXACT("Diets") OR diet OR dietary OR MAINSUBJECT.EXACT("Eating Behavior") OR MAINSUBJECT.EXACT("Exercise") OR MAINSUBJECT.EXACT("Health Behavior") OR MAINSUBJECT.EXACT("Lifestyle") OR lifestyles OR MAINSUBJECT.EXACT("Lifestyle Changes") OR MAINSUBJECT.EXACT("Nutrition") OR MAINSUBJECT.EXACT("Physical Activity") OR "physical activities" OR MAINSUBJECT.EXACT("Physical Fitness") OR MAINSUBJECT.EXACT("Sedentary Behavior") OR sitting OR MAINSUBJECT.EXACT.EXPLODE("Weight Control") OR MAINSUBJECT.EXACT("Weight Loss" OR "weight maintenance") |
| 3 | #1 AND #2  Limited by Record Type: Peer-reviewed Journals; Dissertations |

**IEEE**

| # | Searches |
| --- | --- |
| 1 | ("Ai agent" OR “Ai agents” OR "Artificial agent" OR "Artificial agents" OR “Artificial intelligence chatbot” OR “Artificial intelligence chatbots” OR "Assistance technology" OR "Assistance technologies" OR “Chat bot”  OR “Chat bots” OR Chatbot OR Chatbots OR Chatterbot OR "Conversational agent" OR "Conversational agents" OR “Conversational AI” OR "Conversational assistant" OR "Conversational assistants" OR “Conversational bot” OR “Conversational bots” OR "Conversational interface" OR "Conversational interfaces" OR "Conversational system" OR "Conversational systems" OR "Dialog system" OR "Dialog systems" OR “Dialogue agent” OR "Dialogue system" OR "Dialogue systems"  OR "Digital assistant" OR "Digital assistants" OR Infobot OR "Intelligent agent" OR "Intelligent agents" OR “Intelligent conversational assistant” OR “Interactive agent” OR “Interactive agents” OR "Relational agent" OR "Relational agents" OR “Social robot” OR “Social robots” OR “Virtual advisors” OR "Virtual agent" OR "Virtual agents" OR “Virtual assistant” OR “Virtual assistants” OR “Virtual avatar” OR “Virtual avatars” OR “Virtual coach” OR “Virtual coaches” OR “Virtual conversational agents”) |
| 2 | (Diet OR Diets OR Dietary OR “eating behavior” OR Exercise OR Exercises OR Lifestyle OR Lifestyles OR “life style” OR “life styles” OR nutrition OR "Physical activity" OR “Physical activities” OR “Sedentary behavior” OR “Sedentary time” OR sitting OR “weight control’ OR “Weight loss” OR “Weight losses” OR “Weight maintenance”) |
| 3 | #1 AND #2  Broken down by conference paper  Broken down by journal article |
