# Supplementary material for: A systematic review of artificial intelligence chatbots for promoting physical activity, healthy diet, and weight loss
Source: Int J Behav Nutr Phys Act. 2021 Dec 11;18:160. doi: 10.1186/s12966-021-01224-6 (PMC8665320; doi:10.1186/s12966-021-01224-6)
Supplement: Supplementary file 2 — Additional file 2. Summary of quality assessment and risk of bias. [file 12966_2021_1224_MOESM2_ESM.docx]

| **Controlled Intervention Studies** | | | | |
| --- | --- | --- | --- | --- |
| **Quality assessment criteria: Yes, No, Other (CD, NR, NA)** | **Publication (First author/Year/Country)** | | | |
|  | Kramer J/ 2020/  Switzerland | Kunzler F/ 2019/  Switzerland | Piao M/ 2020/  South Korea | Carfora V/ 2019/  Italy |
| 1. Was the study described as randomized, a randomized trial, a randomized clinical trial, or an RCT? | Yes | Yes | Yes | Yes |
| 2. Was the method of randomization adequate (i.e., use of randomly generated assignment)? | Yes | Yes | Yes | Yes |
| 3. Was the treatment allocation concealed (so that assignments could not be predicted)? | Yes | Yes | Yes | Yes |
| 4. Were study participants and providers blinded to treatment group assignment?^1)^ | NA | NA | NA | NA |
| 5. Were the people assessing the outcomes blinded to the participants' group assignments? | NR | NR | NR | NR |
| 6. Were the groups similar at baseline on important characteristics that could affect outcomes (e.g., demographics, risk factors, co-morbid conditions)? | NR | NR | Yes | Yes |
| 7. Was the overall drop-out rate from the study at endpoint 20% or lower of the number allocated to treatment? | No | NR | Yes | Yes |
| 8. Was the differential drop-out rate (between treatment groups) at endpoint 15 percentage points or lower? | Yes | NR | No | NR |
| 9. Was there high adherence to the intervention protocols for each treatment group? | No | NR | Yes | Yes |
| 10. Were other interventions avoided or similar in the groups (e.g., similar background treatments)? | NR | NR | NR | NR |
| 11. Were outcomes assessed using valid and reliable measures, implemented consistently across all study participants? | Yes | Yes | Yes | Yes |
| 12. Did the authors report that the sample size was sufficiently large to be able to detect a difference in the main outcome between groups with at least 80% power? | Yes | No | Yes | Yes |
| 13. Were outcomes reported or subgroups analyzed prespecified (i.e., identified before analyses were conducted)? | Yes | No | Yes | Yes |
| 14. Were all randomized participants analyzed in the group to which they were originally assigned, i.e., did they use an intention-to-treat analysis? | Yes | Yes | No | No |
| **Quality Rating (Good, Fair, or Poor; If Poor, please state why)** | **Fair** | **Poor** | **Fair** | **Fair** |

**Additional file 2. Summary of quality assessment and risk of bias**

Note. CD=cannot determine; NA=not applicable; NR=not reported

1) Blinding was considered as “not applicable” in chatbot-based behavioral change interventions

| **Before-After (Pre-Post) Studies With No Control Group** | | | | | |
| --- | --- | --- | --- | --- | --- |
| **Quality assessment criteria: Yes, No, Other (CD, NR, NA)** | **Publication (First author/Year/Country)** | | | | |
|  | Maher CA/ 2020/  Australia | Fadhil A/ 2019/  NR | Stephens TN/ 2019/  U.S. | Casas J/  2018/  Switzerland | Kocielnik R/  2018/  U.S. |
| 1. Was the study question or objective clearly stated? | Yes | Yes | Yes | Yes | Yes |
| 2. Were eligibility/selection criteria for the study population prespecified and clearly described? | Yes | No | Yes | No | No |
| 3. Were the participants in the study representative of those who would be eligible for the test/service/intervention in the general or clinical population of interest? | No | No | No | No | No |
| 4. Were all eligible participants that met the prespecified entry criteria enrolled? | Yes | No | Yes | NR | NR |
| 5. Was the sample size sufficiently large to provide confidence in the findings? | Yes | No | No | No | No |
| 6. Was the test/service/intervention clearly described and delivered consistently across the study population? | Yes | Yes | Yes | Yes | Yes |
| 7. Were the outcome measures prespecified, clearly defined, valid, reliable, and assessed consistently across all study participants? | Yes | Yes | Yes | No | Yes |
| 8. Were the people assessing the outcomes blinded to the participants' exposures/interventions? | No | No | No | No | No |
| 9. Was the loss to follow-up after baseline 20% or less? Were those lost to follow-up accounted for in the analysis? | Yes | NR | NR | NR | NR |
| 10. Did the statistical methods examine changes in outcome measures from before to after the intervention? Were statistical tests done that provided p values for the pre-to-post changes? | Yes | Yes | No | No | Yes |
| 11. Were outcome measures of interest taken multiple times before the intervention and multiple times after the intervention (i.e., did they use an interrupted time-series design)? | No | No | No | No | No |
| 12. If the intervention was conducted at a group level (e.g., a whole hospital, a community, etc.) did the statistical analysis take into account the use of individual-level data to determine effects at the group level?^1)^ | NA | NA | NA | NA | NA |
| **Quality Rating (Good, Fair, or Poor; If Poor, please state why)** | **Fair** | **Poor** | **Poor** | **Poor** | **Poor** |

1) Group-level analysis was considered as “not applicable” in chatbot-based behavioral change interventions
